# Supplementary material for: Dysregulated Neutrophil Phenotype and Function in Hospitalised Non-ICU COVID-19 Pneumonia
Source: Cells. 2022 Sep 16;11(18):2901. doi: 10.3390/cells11182901 (PMC9496854; doi:10.3390/cells11182901)
Supplement: Supplementary file 1 [file cells-11-02901-s001.zip › Supplementary Figures.pdf]

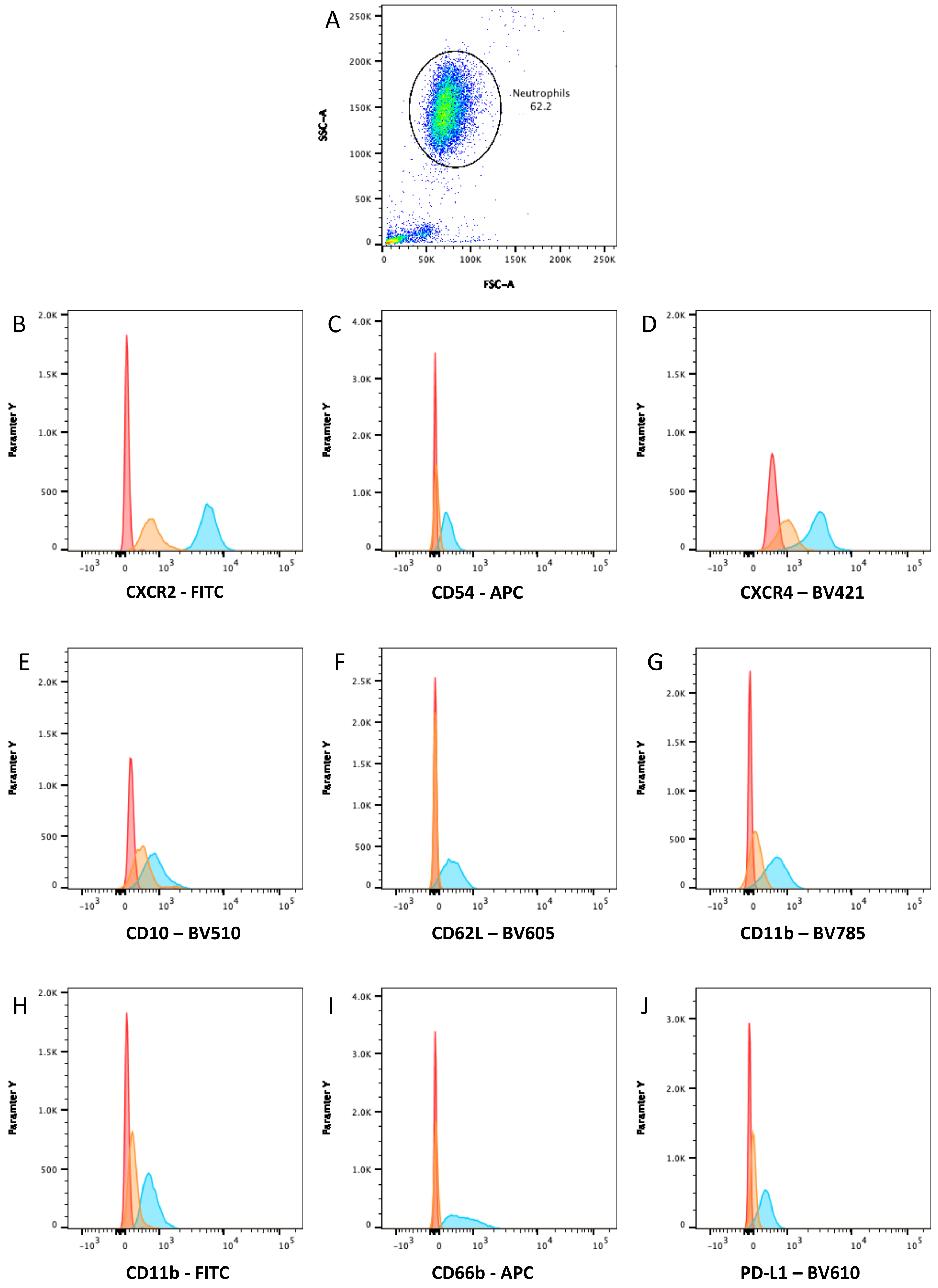

Supplementary Figure S1 – Flow cytometry gating strategy for receptor expression. Neutrophils were gated on forward vs. side scatter. Expression of receptors was analysed within this gate. Histograms show negative unstained control in red, isotype control in orange, and positive sample in blue.

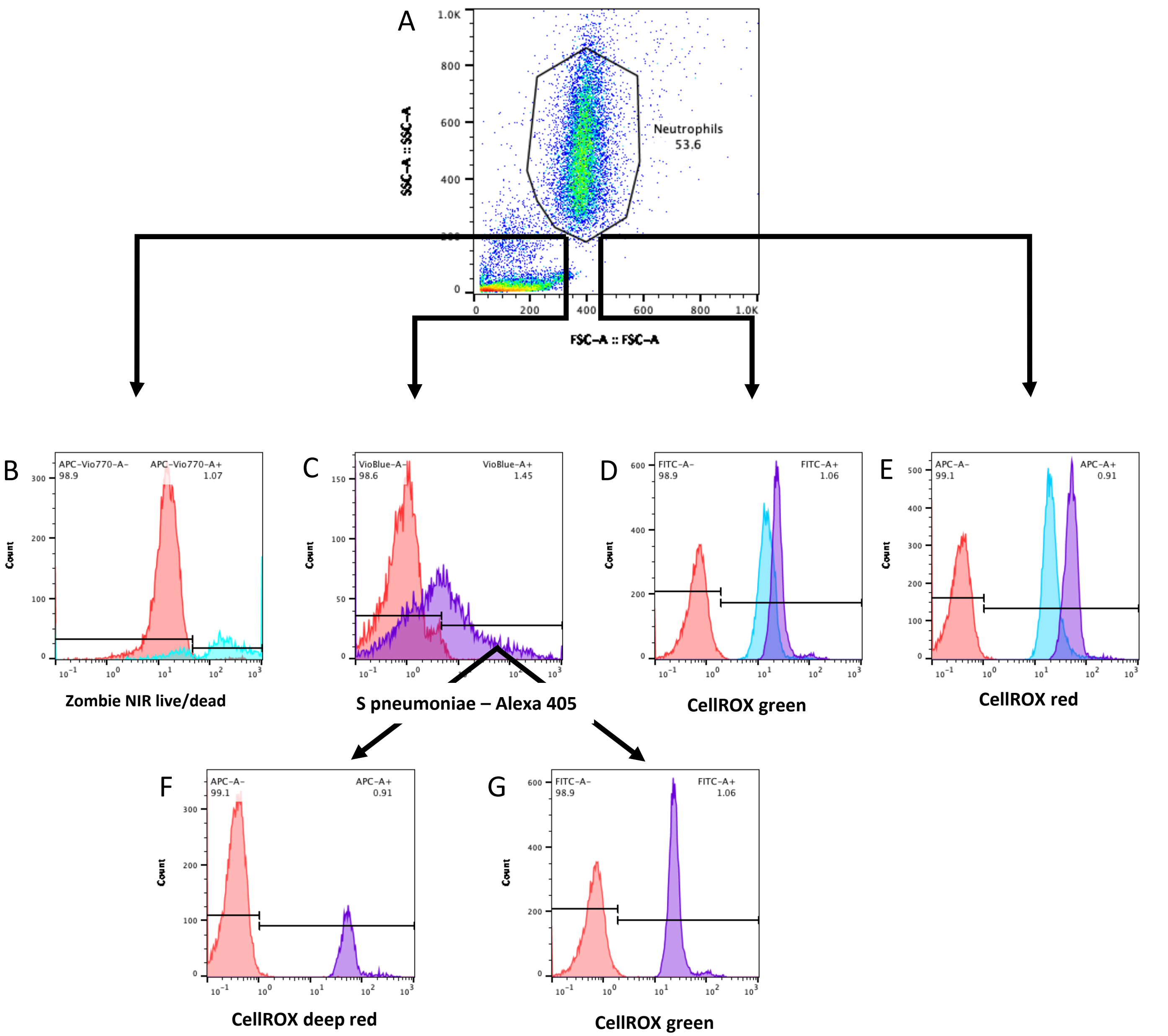

Supplementary Figure S2 – Flow cytometry gating strategy for functional output. Neutrophils were gated on forward vs. side scatter (A). Within this population, multiple parameters were measured: B) Zombie NIR live/dead. C) Phagocytosis of Alexa-405 labelled S. pneumoniae, D) CellROX green, E) CellROX red. Within the SP+ gate, both CellROX deep red (F) and CellROX green (G) were measured.

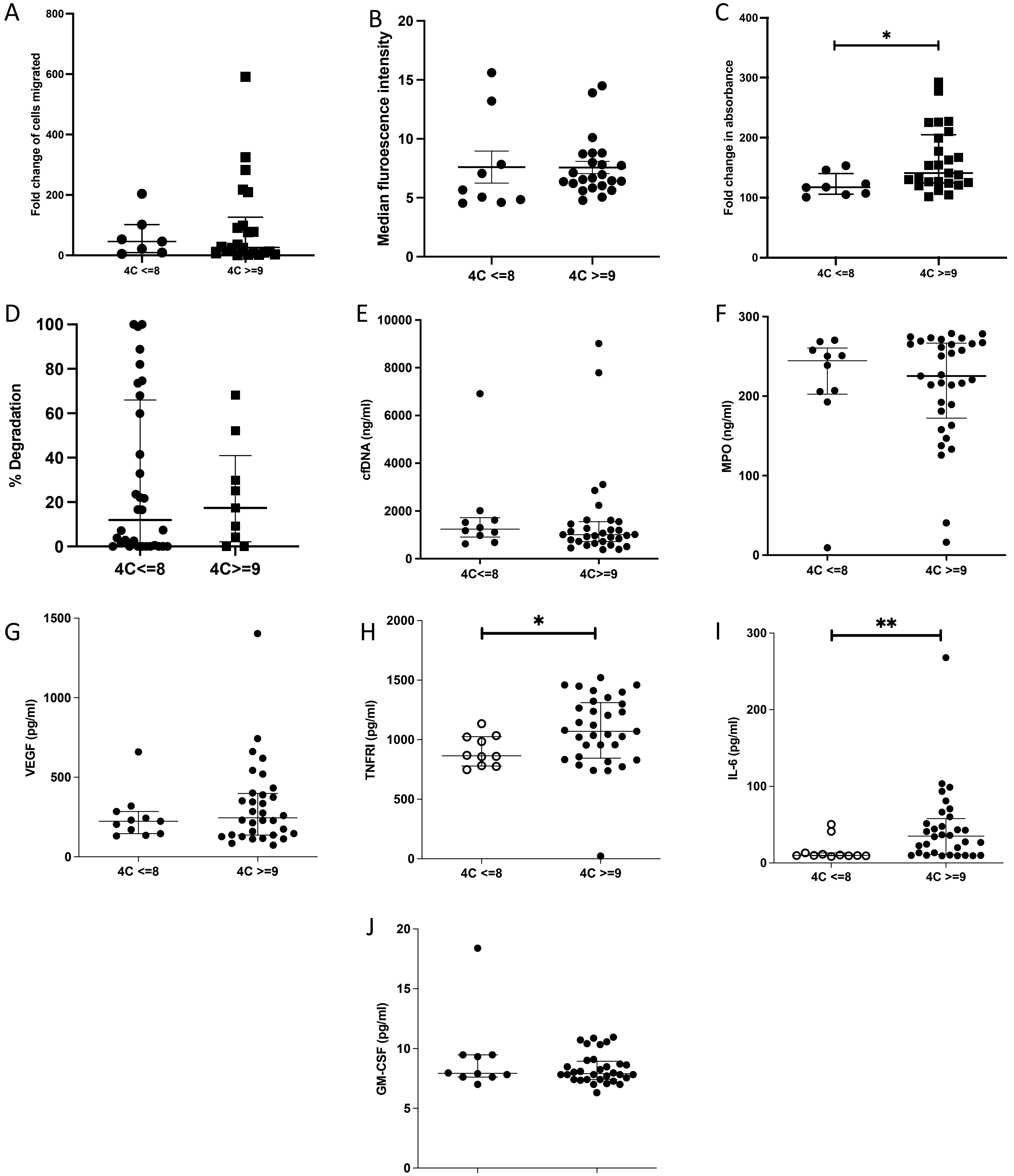

Supplementary Figure S3 – 4C severity score analysis, analysed by Mann-Whitney test. A) Neutrophil transmigration towards CXCL-8. Data shows fold change in neutrophils migrated to CXCL-8 compared to vehicle control. 4C<=8 (n=7), 4C>=9 (n=23), p=0.5575. B) Neutrophil phagocytosis of opsonised S. pneumoniae following a 30-minute incubation. Data shows MFI of positive neutrophils. 4C<=8 (n=9), 4C>=9 (n=23), p=0.3257. C) NET release by neutrophils stimulated with PMA for 3 hours. Data shows fold change in absorbance of PMA stimulated neutrophils compared to vehicle control. 4C<=8 (n=8), 4C>=9 (n=25), *p=0.0118. D). % DNA degradation by serum NETs, 4C<=8 (n=9), 4C>=9 (n=32), p=0.6054. E) Plasma cell free DNA levels, 4C<=8 (n=10), 4C>=9 (n=31), p=0.3001. F) Plasma MPO levels. 4C<=8 (n=9), 4C>=9 (n=32), p=0.9436. G) Plasma VEGF levels, 4C<=8 (n=9), 4C>=9 (n=32), p=0.5961. H) Plasma TNFRI levels, 4C<=8 (n=9), 4C>=9 (n=32), *p=0.0478 I) Plasma IL-6 levels, 4C<=8 (n=9), 4C>=9 (n=32), *p=0.0059. J) Plasma GM-CSF levels, 4C<=8 (n=9), 4C>=9 (n=32), p=0.6049. Data expressed as median±IQR.

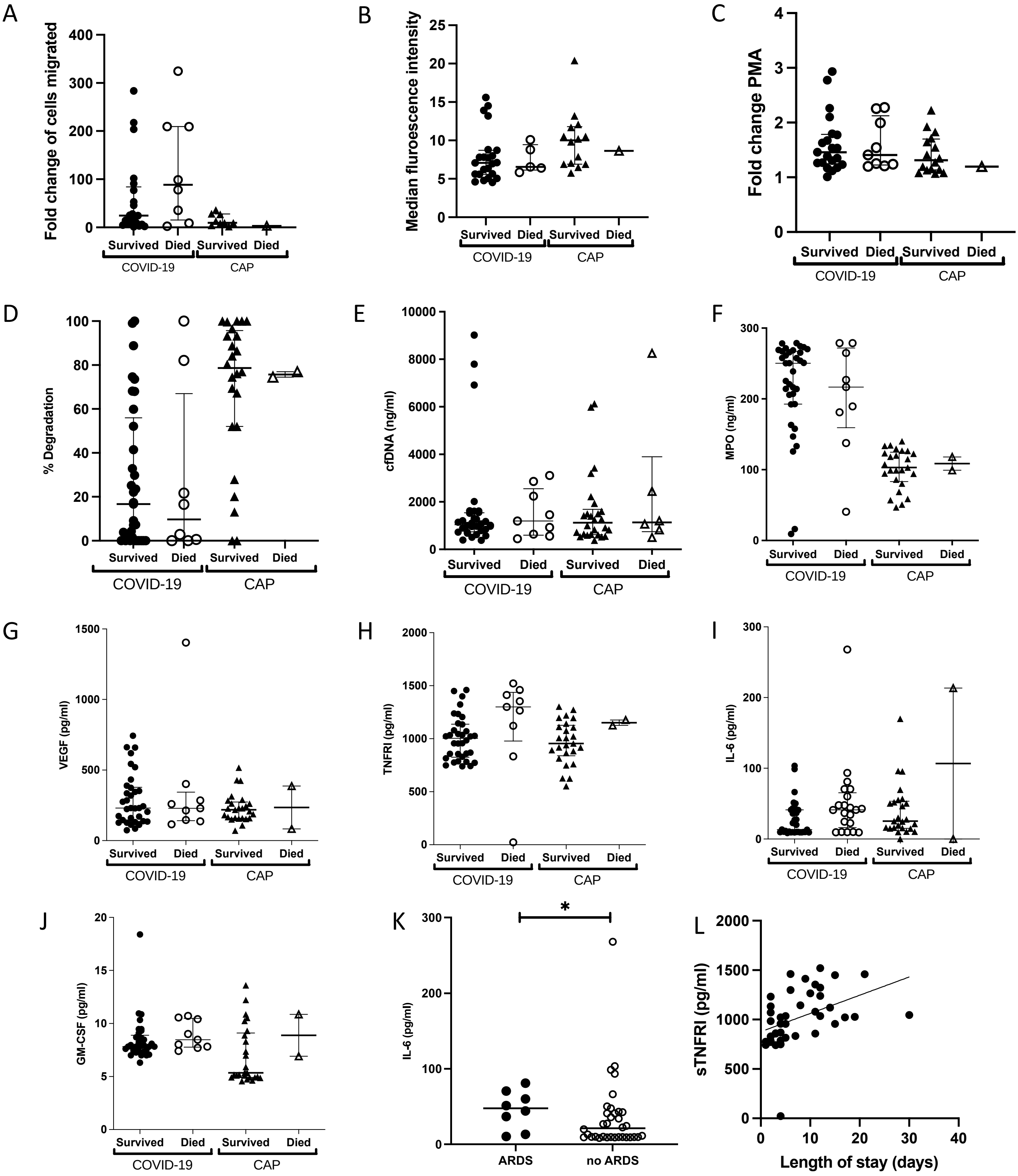

A
Fold change of cells migrated
0
100
200
300
400
Survived
Died
Survived
Died
COVID-19
CAP
B
Median fluroescence intensity
0
5
10
15
20
25
Survived
Died
Survived
Died
COVID-19
CAP
C
Fold change PMA
0
1
2
3
4
Survived
Died
Survived
Died
COVID-19
CAP
D
% Degradation
0
20
40
60
80
100
Survived
Died
Survived
Died
COVID-19
CAP
E
cfDNA (ng/ml)
0
2000
4000
6000
8000
10000
Survived
Died
Survived
Died
COVID-19
CAP
F
MPO (ng/ml)
0
100
200
300
Survived
Died
Survived
Died
COVID-19
CAP
G
VEGF (pg/ml)
0
500
1000
1500
Survived
Died
Survived
Died
COVID-19
CAP
H
TNFRI (pg/ml)
0
500
1000
1500
2000
Survived
Died
Survived
Died
COVID-19
CAP
I
IL-6 (pg/ml)
0
100
200
300
Survived
Died
Survived
Died
COVID-19
CAP
J
GM-CSF (pg/ml)
0
5
10
15
20
Survived
Died
Survived
Died
COVID-19
CAP
K
IL-6 (pg/ml)
*
0
100
200
300
ARDS
no ARDS
L
sTNFRI (pg/ml)
0
500
1000
1500
2000
0
10
20
30
40
Length of stay (days)

Supplementary Figure S4 – Survival analysis, analysed by One way ANOVA. A) Neutrophil transmigration towards IL-8. Data shows fold change in neutrophils migrated to CXCL-8 compared to vehicle control. COVID-19 - Survived (n=23), died (n=8), p=0.2563, CAP – survived (N=9), died (n=1), p=0.9039. B) Neutrophil phagocytosis of opsonised S. pneumoniae following a 30-minute incubation. Data shows MFI of positive neutrophils. COVID-19 - Survived (n=26), died (n=7), p=0.9228. CAP – survived (n=14), died (n=1), p=0.998 C) NET release by neutrophils stimulated with PMA for 3 hours. Data shows fold change in absorbance of PMA stimulated neutrophils compared to vehicle control. COVID-19 - survived (n=21), died (n=9), p=0.999, CAP – survived (n=16), died (n=1), p=0.999. D). % DNA degradation by serum NETs, COVID-19 - survived (n=33), died (n=8), p=0.9895, CAP – survived (n=20), died (n=4), p=0.995. E) Plasma cell free DNA levels, COVID-19 - survived (n=32), died (n=9), p=0.8406, CAP – survived (N=23), died (n=2), p=0.956. F) Plasma MPO levels. Survived (n=34), died (n=9), p=0.6888. G) Plasma VEGF levels Survived (n=34), died (n=9), p=0.9924. H) Plasma TNFRI levels, Survived (n=34), died (n=9), *p=0.0311 I) Plasma IL-6 levels, Survived (n=34), died (n=9), p=*0.0027. J) Plasma GM-CSF levels, Survived (n=34), died (n=9), p=0.1875. K) Plasma IL-6 levels in patients with ARDS (n=8) or no ARDS (N=34), p=0.0468. L) Correlation of length of hospital day vs. sTNFRI plasma levels. p=0.006, r^2=0.1781. Data expressed as median±IQR.

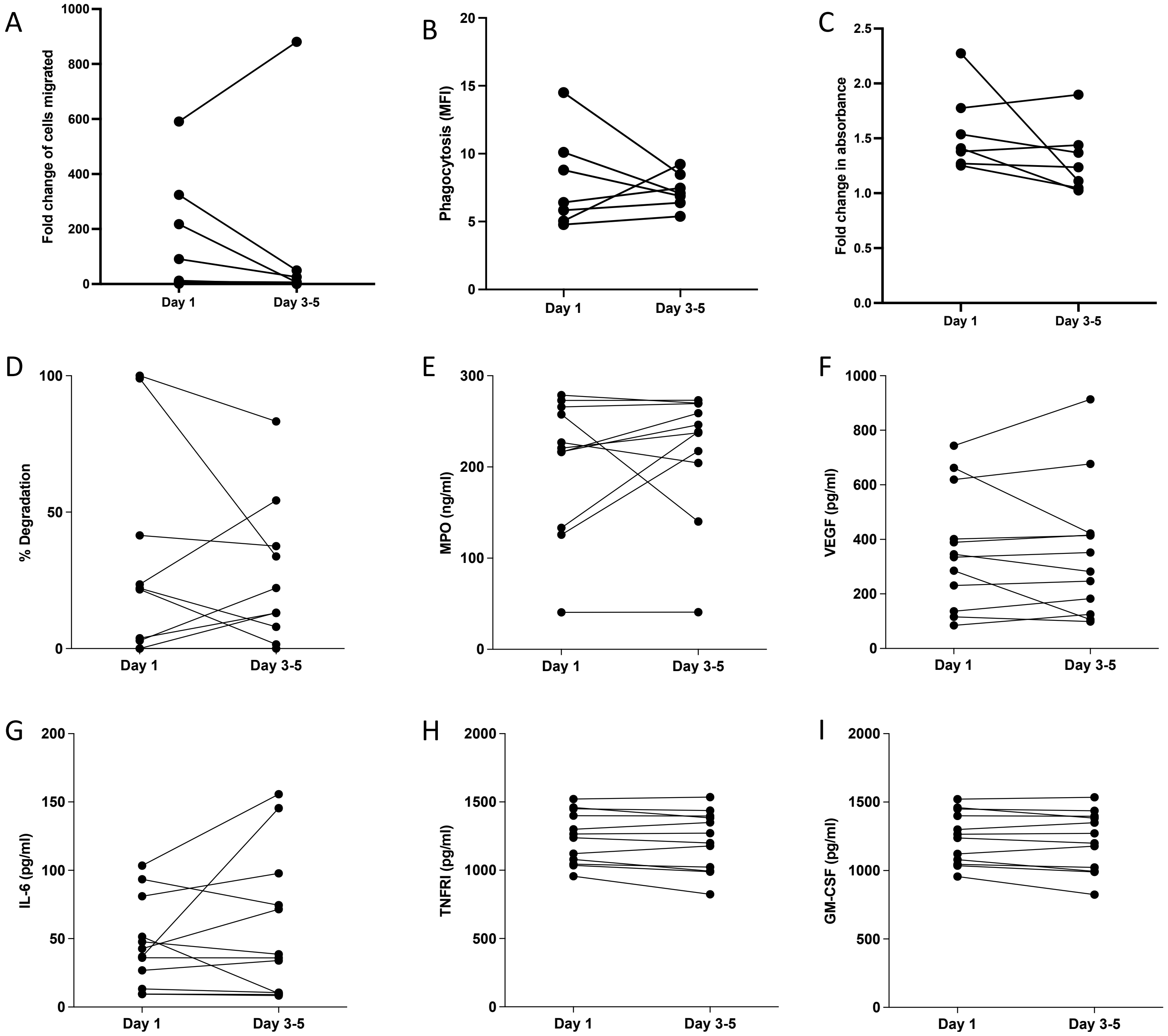

Supplementary Figure S5 – Follow up sample analysis, analysed by Wilcoxon test. A) Neutrophil transmigration towards 100nM CXCL-8. Data shows fold change in neutrophils migrated to CXCL-8 compared to vehicle control. N=6, p=0.1641. B) Neutrophil phagocytosis of opsonised S. pneumoniae following a 30-minute incubation. Data shows MFI of positive neutrophils. N=6, p=0.8125. C) NET release by neutrophils stimulated with25nm PMA for 3 hours. Data shows fold change in absorbance of PMA stimulated neutrophils compared to vehicle control. N=7, p=0.1562. D). % DNA degradation by serum NETs, n=10, p=0.7544. E) Plasma MPO levels. N=11, p=0.6063. F) Plasma VEGF levels, n=12, p=0.999. G) Plasma TNFRI levels, n=12, p=0.7125 H) Plasma IL-6 levels, n=12, p=0.9647. I) Plasma GM-CSF levels, n=12, p=0.7125. Data expressed as median±IQR.

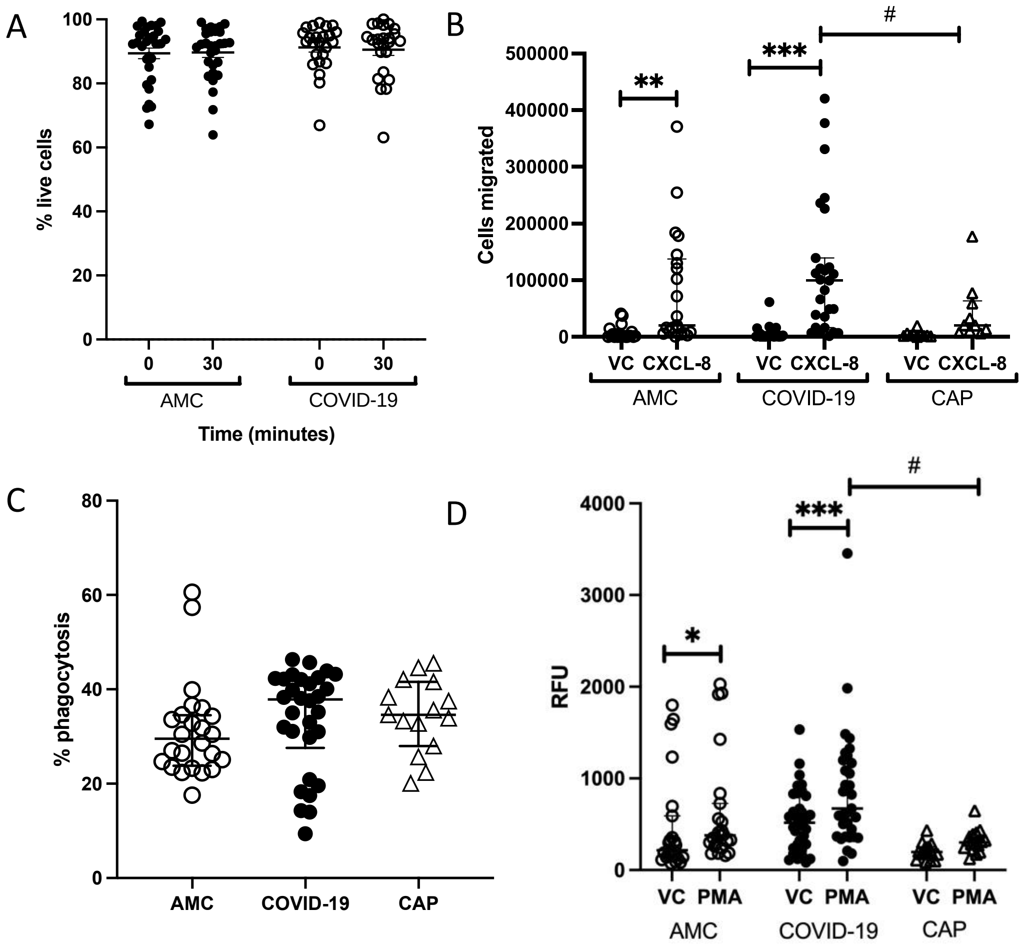

Supplementary Figure S6 – A) Viability of isolated neutrophils at baseline (0) and after phagocytosis (30 minutes) in AMC (n=24) and COVID-19 patients (n=30), measured by Zombie NIR viability stan. Data analysed by two way ANOVA. p=0.6726. B) Number of cells migrated towards 100nM CXCL-8 using transwell chambers, showing vehicle control (VC) or CXCL-8. AMC n=18, COVID-19 n=28, CAP n=10. Analysed by two-way ANOVA. **p<0.0038, ***p<0.0001 VC vs CXCL-8. #p=0.0223 COVID-19 vs. CAP. C) Percentage of neutrophils that phagocytosed *S. pneumoniae* in AMC (n=24), COVID-19 patients (n=30) and CAP patients (n=15). p=0.05666, analyzed by Mann-Whitney test. D) NETosis by isolated neutrophils, showing absorbance values of cfDNA (relative fluorescence units (RFU), showing vehicle control (VC) or 25nM PMA. AMC n=23, COVID-19 n=33, CAP n=16. Analyzed by two-way ANOVA. **p<0.0206, ***p<0.0001 VC vs PMA. #p=0.009 COVID-19 vs CAP.

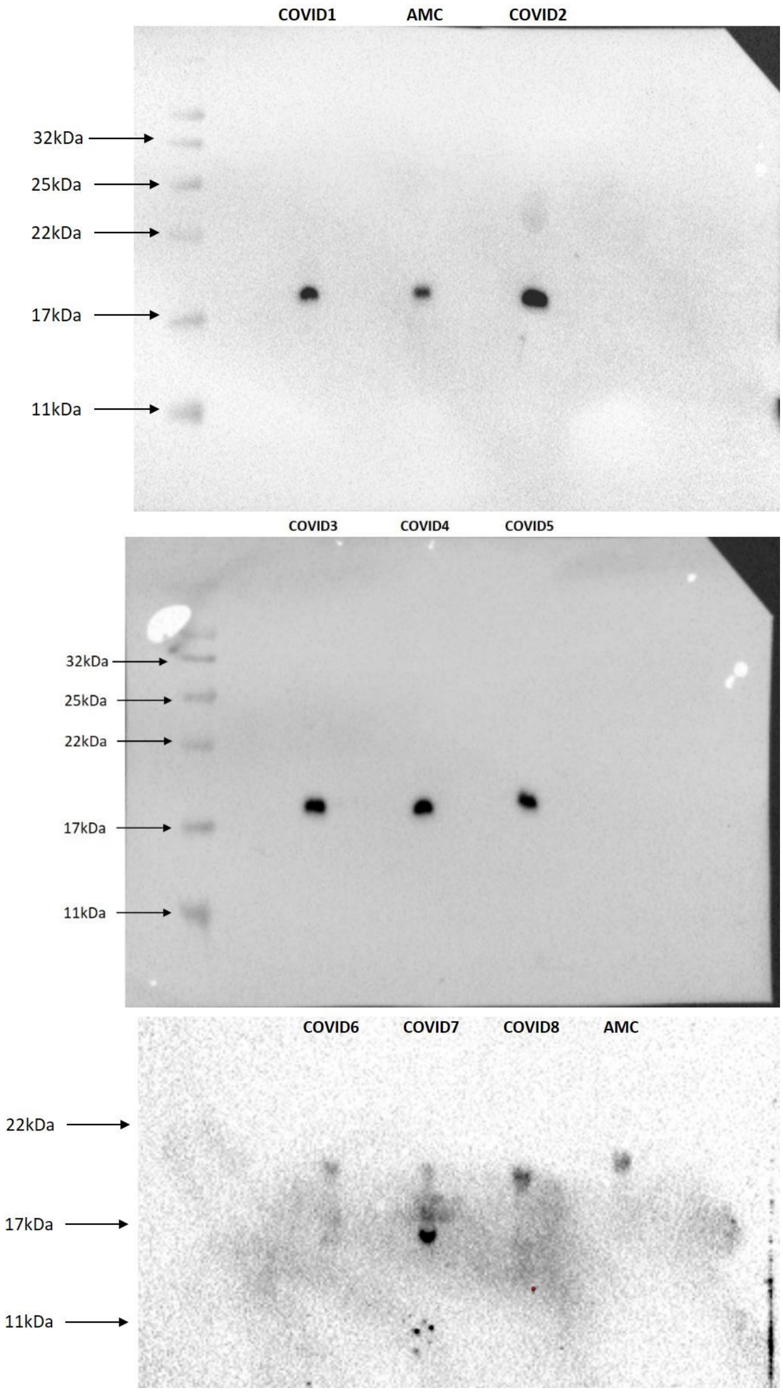

Supplementary Figure S7 – Detection of citrulinated histone 3 (CitH3) in plasma samples from COVID-19 patients and age matched controls. Plasma samples were electrophoresed and blotted for CitH3 (Abcam). Molecular weight markers are indicated with arrows.

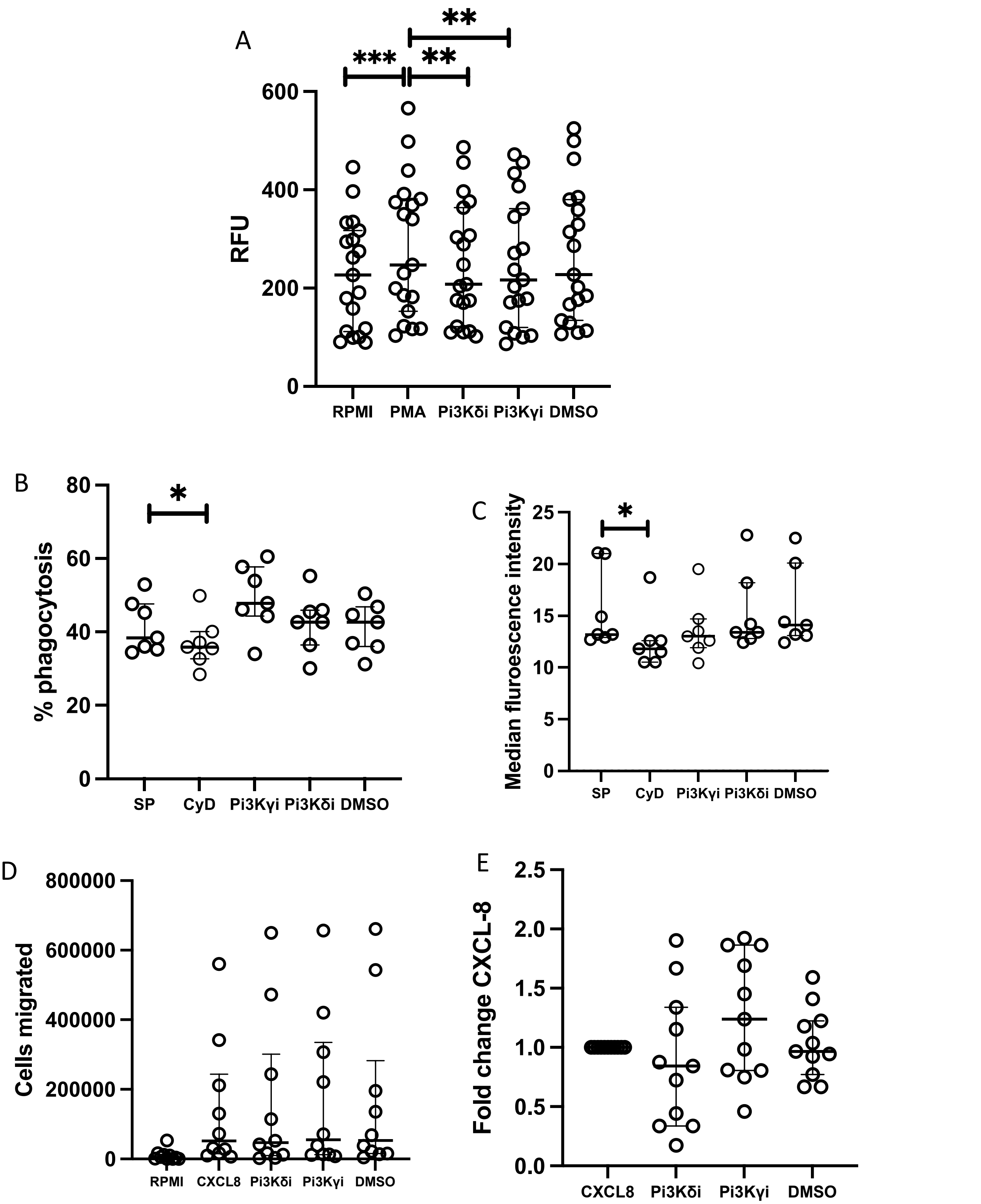

Supplementary Figure S8 – Effect of PI3K inhibitors on neutrophil function. A) NETosis by isolated neutrophils, showing absorbance values of cfDNA (relative fluorescence units (RFU), showing RPMI control, 25nM PMA, or treatments + PMA. (n=19) ***p<0.001 RPMI vs. PMA. **p=0.0021 PMA vs. PI3K**δ**I, **p=0.0091 PMA vs. PI3K**γ**I. B) % neutrophil phagocytosis of *S. pneumoniae* (n=7). *p=0.0495 SP vs. Cytochalasin D. C) Median fluorescence intensity of phagocytosis (n=7). P=0.0432 SP vs. CytoD. D) Transwell migration to CXCL-8, showing RPMI control, CXCL-8, or treatments + CXCL-8 (n=11) p=0.0520. E) Fold change in migration to CXCL-8 (n=11), p=0.2245.
